# CRP levels are significantly associated with CRP genotype and estrogen use in The Lifestyle, Biomarker and Atherosclerosis (LBA) study

**DOI:** 10.1186/s12872-022-02610-z

**Published:** 2022-04-15

**Authors:** Karin Fransén, Carolina Pettersson, Anita Hurtig-Wennlöf

**Affiliations:** 1 School of Medical Sciences, Örebro University, 701 82 Örebro, Sweden; 2 Clinical Research Laboratory, Faculty of Medicine and Health, Örebro University, Örebro, Sweden; 3 School of Health and Welfare, Jönköping University, Jönköping, Sweden

**Keywords:** Atherosclerosis, cIMT, Cardiovascular risk, C-reactive protein, CRP, Estrogen, Genotype, Polymorphism, SNP, Young adults

## Abstract

**Background:**

The C-reactive protein (CRP) is an important biomarker for atherosclerosis and single nucleotide polymorphisms (SNPs) in the *CRP* locus have been associated with altered CRP levels and associated with risk for cardiovascular disease. However, the association between genetic variations in the *CRP* gene, estrogen use and CRP levels or early signs of atherosclerosis in young healthy individuals is not fully characterized. We aimed to evaluate the influence of five genetic variants on both plasma CRP levels and carotid intima-media thickness (cIMT) values, including aspects on estrogen containing contraceptive use in females.

**Methods:**

Genotyping was performed with TaqMan real time PCR and compared with high sensitivity CRP serum levels in 780 Swedish young, self-reported healthy individuals. Haplotypes of the SNPs were estimated with the PHASE v 2.1. The cIMT was measured by 12 MHz ultrasound. The contraceptive use was self-reported.

**Results:**

Strong associations between CRP and genotype were observed for rs3091244, rs1800947, rs1130864, and rs1205 in women (all *p* < 0.001). In men, only rs1800947 was associated with CRP (*p* = 0.029). The independent effect of genotypes on CRP remained significant also after adjustment for established risk factors. Female carriers of the H1/ATGTG haplotype had higher CRP than non-carriers. This was specifically pronounced in the estrogen-using group (*p* < 0.001), and they had also higher cIMT (*p* = 0.002) than non-carriers but with a small cIMT difference between the haplotype groups (0.02 mm). In parallel, a significant correlation between CRP and cIMT in the estrogen using group was observed (*r* = 0.194; *p* = 0.026).

**Conclusions:**

Estrogen use, genotypes and haplotypes in the CRP locus are significantly associated with CRP levels. Based on an observed interaction effect between sex/estrogen use and the H1/ATGTG haplotype on CRP, and a marginally thicker cIMT in the estrogen using group, our data suggest that both genotypes and estrogen usage could be involved in arterial wall structural differences. The causality between CRP levels and cIMT remains unclear, and the observed difference in cIMT is not clinically relevant in the present state. Future larger and longitudinal studies may shed further light on the role of more long-term estrogen use and early atherosclerosis.

**Supplementary Information:**

The online version contains supplementary material available at 10.1186/s12872-022-02610-z.

## Background

Atherosclerosis is the major cause of cardiovascular disease (CVD) and is an inflammatory disease of the large and medium-sized arteries. Although CVD is uncommon in young individuals, the atherosclerotic process is initiated early in life. Arterial fatty streaks typically evolve over long time and remains asymptomatic through decades [1]. The formation of fatty streaks is associated with endothelial dysfunction and expression of adhesion molecules, followed by elevated vascular inflammation accumulation of lipids, cholesterol and calcium, foam cell formation, obstruction and finally risk for ischemic event [2, 3].

Due to the long-time progression of atherosclerosis development, the need for early biomarkers and identification of risk factors in young population is warranted. One of the acute-phase reactants, C-reactive protein (CRP), has been pointed out as an important biomarker for atherosclerosis and acute myocardial infarction [4–6]. The CRP protein is involved in the inflammatory response and is activated by the cytokines interleukin (IL)-6 and IL-1β [7]. CRP is a member of the calcium-dependent ligand-binding pentraxin family which circulates in plasma and is expressed in atherosclerotic lesions [8–10]. CRP can also activate the complement system and co-localizes with the membrane attack complex (MAC) in atherosclerotic plaques [8–10]. In addition, CRP regulates the expression of adhesion molecules in the endothelium, which suggest that CRP promotes inflammation in the atherosclerotic lesion [11].

In young healthy individuals, CRP levels are generally low. Elevated levels of CRP in the childhood are significantly associated with higher CRP levels in adults [12]. The CRP protein is encoded by the *CRP* gene, which is located on the human chromosome 1q23.2 and consists of two exons separated by one intron [13–15]. Studies have reported that plasma CRP concentration is under genetic control and in twins a heritability of CRP levels of approximately 50% have been observed [16]. Several *CRP* gene single nucleotide polymorphisms (SNPs) influence the plasma CRP levels in CVDs and previous studies have aimed to elucidate the role of SNPs in the CRP locus, CRP levels and early signs of atherosclerosis [17–22]. Eklund et al. showed a significant association between the *CRP* SNPs rs3091244, rs1130864 or rs1205 in boys and carotid artery compliance (CAC), while Kettunen et al. found a significant association between the SNP rs1130864 and one of the three carotid artery elasticity (CAE) parameters in men [21, 22]. However, neither Eklund or Kettunen found any association between CRP genotype and the carotid intima-media thickness (cIMT) [21, 22]. Contradictory results exist regarding the role of childhood CRP measurements and association with cIMT in the adult [12, 23]. We and others have previously also shown that use of estrogen containing contraceptives are associated with higher CRP levels [24, 25]. The association between genetic variations in the *CRP* gene, estrogen use and CRP levels or preclinical signs of atherosclerosis is however not fully known.

The aim of the present study was therefore to evaluate the influence of common genetic variants and estrogen on plasma CRP levels and carotid intima media thickness (cIMT) values in young, self-reported healthy, individuals in the Swedish Lifestyle, Biomarkers and Atherosclerosis (LBA) cohort, which consists of 834 healthy individuals 18–25.9 years [26]. We selected five different SNPs within the *CRP* gene (rs2794521 (−717A>G), rs3091244 (−286C>T>A), rs1800947 (+1059G>C), rs1130864 (+1444C>T) and rs1205 (+ 1846G>A)), which have previously been reported to influence CRP levels or risk for CVD [17, 21, 22]. These SNPs were also previously included in two Finnish studies on markers of early atherosclerosis [21, 22]. However, in the LBA cohort used in the present study, the participants were non-smokers, and we have included information on self-reported estrogen use, which both are important factors for CRP levels [24, 25].

## Methods

### Study population and ethics

Blood samples from 834 Swedish young, self-reported healthy individuals (18–25.9 yrs) were collected in the Lifestyle, Biomarkers and Atherosclerosis (LBA) cohort, Örebro, Sweden. Details about the LBA cohort and sampling were published in [26]. In the present study, exclusion criteria were CRP level ≥ 10 mg/L (*n* = 36), indicating ongoing inflammation or incomplete haplotype (*n* = 30). Twelve of the individuals with incomplete haplotype also had CRP level ≥ 10 mg/L, which resulted in a total study population of *n* = 780 individuals. Self-report on medication use, including contraceptives, was given by questionnaire, details are described in [23]. Based on the responses, females were grouped into estrogen-containing contraceptive users (*N* = 133; hereafter called “estrogen users” or “EU”) and non-estrogen containing contraceptive users (*N* = 399; “non-estrogen users” or “NEU”).

### DNA extraction

Genomic DNA was isolated with Wizard Genomic DNA purification kit (Promega, Madison WI) according to the supplier’s recommendations. DNA concentration was measured with NanoDrop 200 spectrophotometer (Thermo Fisher Scientific, Waltham, MA) and diluted to 20 ng/µl.

### Genotyping

Four biallelic polymorphisms in the *CRP* gene (rs2794521 (-717 A>G), rs1800947 (+1059 G>C), rs1130864 (+1444 C>T) and rs1205 (+1846 G>A)) and one triallelic polymorphism (rs3091244 (-286 C>T>A) were selected for genotyping. The rs2794521 and the rs3091244 polymorphisms are located in the promotor region of the CRP gene, the rs1800947 is in exon 2 and the two additional polymorphisms rs1130864 and rs1205 are in the 3’ untranslated region. For the biallelic polymorphisms, ten ng of DNA was amplified in a 10 μl reaction containing 1 × TaqMan Genotyping Mastermix (Applied Biosystems, Foster City, CA) and 1 × TaqMan SNP Genotyping Assay (Applied Biosystems) with predesigned primers and probes according to a TaqMan standard protocol in a 7900HT Fast Real-Time PCR system (Applied Biosystems) followed by allelic discrimination analysis. Four reactions in each 96-well plate contained no-template control. The biallelic polymorphisms were displayed in an additive model, except for the biallelic SNP rs1800947, which was displayed in a recessive model due to low amount of homozygous CC individuals.

For the triallelic rs3091244 (-286 C>T>A) polymorphism, two different custom-made genotyping assays containing primers and probes targeting either CA or CT variants were constructed according to [27] and amplified as for the biallelic polymorphisms. A selection of samples from each genotype of the rs3091244 polymorphism were sequenced via Eurofins Genomics sequencing services (www.eurofinsgenomics.eu) to confirm the correct genotyping. Genotypes for the triallelic rs3091244 polymorphism were displayed according [19], with the assumption that the minor A and T alleles have the similar impact on CRP levels [28].

A random selection of 10% of all samples were re-genotyped for each of the five genotypes in a new PCR reaction to verify the accuracy of the genotyping.

### High sensitivity CRP assay and additional serum biomarker analyses

In brief, CardioPhase™High Sensitivity C-Reactive (hsCRP) Assay, ADVIA 1800 Chemistry System (Siemens Healthcare AB, Solna, Sweden) was utilized for CRP analyses. Detailed description of additional serum biomarker analysis was described in [24].

### Haplotype analysis

Haplotypes of the five SNPs were estimated with the PHASE v 2.1 [29, 30]. The SNPs were displayed in the 5’-3’ order, i.e. rs2794521, rs3091244, rs1800947, rs1130864 and rs1205. Haplotypes and haplotype pairs were named according to [21] for haplotypes and haplotype pairs with a frequency > 5%.

### Ultrasound measurements

The cIMT was measured using a high-resolution ultrasound B-mode system, (GE Healthcare, Vivid E9, Chicago, Illinois, US) with a 12 MHz linear array transducer, as previously described [26]. Briefly, the right common carotid artery was scanned and the cIMT was measured over 10 mm in the longitudinal view on the far wall and 10 mm proximal to the carotid bulb, according to guidelines [31].

### Statistical analysis

Statistical analysis was performed using IBM SPSS Statistics for Windows version 27 (IBM Corp, Armonk, NY, USA). Categorical data (sex, genotype, haplotype, and contraceptive use) were reported as frequencies and percentage. Genotype frequencies were evaluated at each SNP locus against those expected in Hardy–Weinberg proportions. Descriptive data on normally distributed variables are presented as mean and standard deviation, and skewed variables (i.e. CRP, triglycerides and insulin) are presented as median and quartiles (Q1-Q3) and ln transformed before further analyses. Correlation coefficients were tested by Pearson’s correlation. To test the difference in CRP levels across genotypes, the Mann Whitney U (if two groups) and Kruskall Wallis tests (if more than two groups) were applied. To explore the association between CRP (dependent) and genotypes, adjusting for earlier described risk factors [32], multiple regression analyses were performed (forced entry). The same set of independent variables were used throughout the testing procedures (i.e. sex, estrogen users/non-estrogen users, body mass index, LDL cholesterol, triglycerides, insulin and, systolic blood pressure). To analyze the main and interaction effects of genotypes, haplotypes, and sex/estrogen use on CRP (dependent), analysis of variance (ANOVA) and analysis of covariance (ANCOVA) were used. In the correlation and inferential statistics, CRP, triglycerides, and insulin were transformed to the natural logarithm (ln) to fit normal distribution and denoted by CRP(ln), triglycerides(ln), and insulin(ln), respectively. Statistical significance level was set to *p* < 0.05.

A posteriori power analysis based on the smallest group and observed standard deviation (i.e. estrogen using women,* n* = 133 and standard deviation = 1.93) showed that a mean difference of CRP = 0.66 mg/L would be able to reject the null hypothesis with a power of 0.80 at the chosen level of significance (i.e. 0.05).

## Results

### Genotype associations

Clinical characteristics are displayed in Table 1 and as expected, there were a significant difference between several of the physiological and biochemical parameters between women and men, including CRP. For the 780 individuals, all of the five polymorphisms were found in Hardy–Weinberg equilibrium. A strong significant association between CRP and genotype was observed for rs3091244, rs1800947, rs1130864 and rs1205 in women (Table 2). In men, only rs1800947 SNP was significantly associated with CRP levels. In women, the minor alleles A and T of the polymorphism rs3091244 (contributing to the genotypes AA + TA + TT) or female individuals homozygous for the minor allele (T) of the SNP rs1130864 were associated with higher median CRP levels. On the other hand, the minor (C) allele (contributing to the genotypes GC and CC), of the rs1800947 polymorphism or female individuals homozygous for the minor (A) allele of the SNP rs1205 were associated with lower median CRP levels (Table 2).

**Table 1:** Clinical characteristics for the 780 individuals in the Lifestyle, Biomarkers and Atherosclerosis (LBA) cohort

	Women (*n* = 532)	Men (*n* = 248)	*p*
Age (years)	21.9 ± 1.9	22.0 ± 2.0	0.193
Height (cm)	168.6 ± 6.4	181.7 ± 6.6	< 0.001
Weight (kg)	63.6 ± 10.9	77.6 ± 11.4	< 0.001
BMI (kg/m^2^)	22.3 ± 3.4	23.5 ± 3.0	< 0.001
Systolic blood pressure (mmHg)	109.8 ± 8.4	122.2 ± 10.9	< 0.001
Diastolic blood pressure (mmHg)	63.9 ± 6.3	64.2 ± 6.7	0.003
Carotid intima-media thickness (mm)	0.49 ± 0.057	0.50 ± 0.064	0.028
CRP median (Q1–Q3) (mg/L)	0.69 (0.34–1.76)	0.55 (0.24–1.16)	< 0.001
Insulin, fasting (mU/L) median (Q1–Q3)	7.11 (5.14–9.79)	6.97 (4.72–9.08)	0.213
Glucose, fasting (mmol/L)	4.93 ± 0.34	5.17 ± 0.33	< 0.001
Triglycerides (mmol/L) Median (Q1–Q3)	0.70 (0.60–1.00)	0.70 (0.60–0.90)	0.956
HDL (mmol/L)	1.45 ± 0.36	1.23 ± 0.28	< 0.001
LDL (mmol/L)	2.30 ± 0.72	2.31 ± 0.69	0.974
Total cholesterol (mmol/L)	4.30 ± 0.77	4.06 ± 0.79	< 0.001
Estrogen contraceptive user (No/yes)	399/133	248/0	

The values are displayed as means (± SD) except for the concentration of C-reactive protein (CRP), fasting insulin and triglycerides, which are presented as median (Q1–Q3) due to skewness. Estrogen contraceptive user displays the frequency (%). The *p*-values are based on Mann–Whitney U test

**Table 2:** Median CRP levels (Q1–Q3) in the LBA cohort in relation to *CRP* gene SNPs

	Women total (*n* = 532)			Women estrogen users (*n* = 133)			Women non-estrogen users (*n* = 399)			Men total (*n* = 248)		
	CRP mg/L (*n*)		*p*	CRP mg/L (*n*)		*p*	CRP mg/L (*n*)		*p*	CRP mg/L (*n*)		*p*
	Median	Q1–Q3		Median	Q1–Q3		Median	Q1–Q3		Median	Q1–Q3	
rs2794521 (−717A>G)												
AA	0.75 (274)	0.36–1.90	0.622	2.22 (77)	0.74–3.38	0.187	0.62 (197)	0.28–1.14	0.894	0.54 (132)	0.27–1.23	0.484
AG	0.67 (215)	0.31–1.63		1.22 (44)	0.65–3.16		0.55 (171)	0.29–1.19		0.52 (103)	0.22–1.05	
GG	0.61 (43)	0.35–1.40		0.90 (12)	0.58–2.20		0.55 (31)	0.30–0.91		0.63 (13)	0.38–1.32	
rs3091244 (−286C>T>A)												
CC	0.55 (211)	0.28–1.06	< 0.001	0.75 (45)	0.48–2.00	< 0.001	0.49 (166)	0.26–0.95	0.032	0.44 (80)	0.22–1.26	0.325
CA + CT	0.81 (253)	0.39.1.96		2.00 (64)	0.95–3.18		0.65 (189)	0.34–1.32		0.52 (122)	0.26–1.02	
AA + TA + TT	1.02 (68)	0.52–3.00		3.12 (24)	1.10–4.53		0.69 (44)	0.38–1.47		0.74 (46)	0.27–1.52	
rs1800947 (+1059G>C)												
GG	0.75 (463)	0.37–1.94	< 0.001	1.77 (120)	0.76–3.24	0.064	0.61 (343)	0.31–1.28	0.002	0.56 (228)	0.26–1.24	0.029
GC + CC	0.47 (69)	0.24–0.79		0.61 (13)	0.51–1.87		0.44 (56)	0.23–0.70		0.26 (20)	0.19–0.94	
rs1130864 (+1444C>T)												
CC	0.59 (256)	0.29–1.18	< 0.001	0.87 (56)	0.53–2.24	< 0.001	0.52 (200)	0.26–0.99	0.076	0.46 (112)	0.22–1.26	0.515
CT	0.84 (222)	0.38–2.14		2.30 (60)	1.04–3.59		0.65 (162)	0.34–1.29		0.57 (107)	0.28–10.2	
TT	1.02 (54)	0.48–2.91		2.89 (17)	1.04–4.37		0.75 (37)	0.36–1.50		0.54 (29)	0.25–1.64	
rs1205 (+1846G>A)												
GG	0.85 (230)	0.42–2.33	< 0.001	2.27 (67)	0.92–4.23	0.011	0.64 (163)	0.34–1.40	0.006	0.56 (120)	0.25–1.26	0.260
GA	0.67 (237)	0.32–1.48		1.36 (49)	0.58–2.52		0.56 (188)	0.29–1.16		0.57 (107)	0.24–1.05	
AA	0.47 (65)	0.23–1.04		0.67 (17)	0.51–2.39		0.36 (48)	0.21–0.89		0.32 (21)	0.18–1.40	

The *p*-values are based on Kruskal Wallis test (if three or more groups) or Mann–Whitney U test (if two groups)

### Genotype associations with CRP in female estrogen and non-estrogen users

Previously, we showed that women in the LBA cohort had significantly higher plasma CRP levels than men and that estrogen using women has significantly higher plasma CRP levels than non-estrogen using women [24, 33]. The women were therefore stratified into non-estrogen users and estrogen users and CRP levels were compared against genotype of the five SNPs (Table 2). The two SNPs rs3091244 and rs1205 were significantly associated with CRP levels even after stratification in non-estrogen users and in estrogen users and thereby independent of estrogen use (EU: *p* < 0.001 and *p* = 0.011 respectively; NEU: *p* = 0.032 and *p* = 0.06 respectively; Table 2). In addition, the rs1130864 polymorphism was strongly significantly associated with altered CRP levels, with higher levels in individuals homozygous for the (T) allele in estrogen users (*p* < 0.001). A similar but non-significant trend of association with higher CRP levels was seen for non-estrogen users (*p* = 0.076; Table 2). Moreover, the rare allele of the SNP rs1800947 was significantly associated with lower CRP levels in non-estrogen users homozygous and heterozygous for the C allele (*p* = 0.002) and a similar but non-significant trend was observed for the estrogen users (*p* = 0.064; Table 2).

**Fig. 1:**
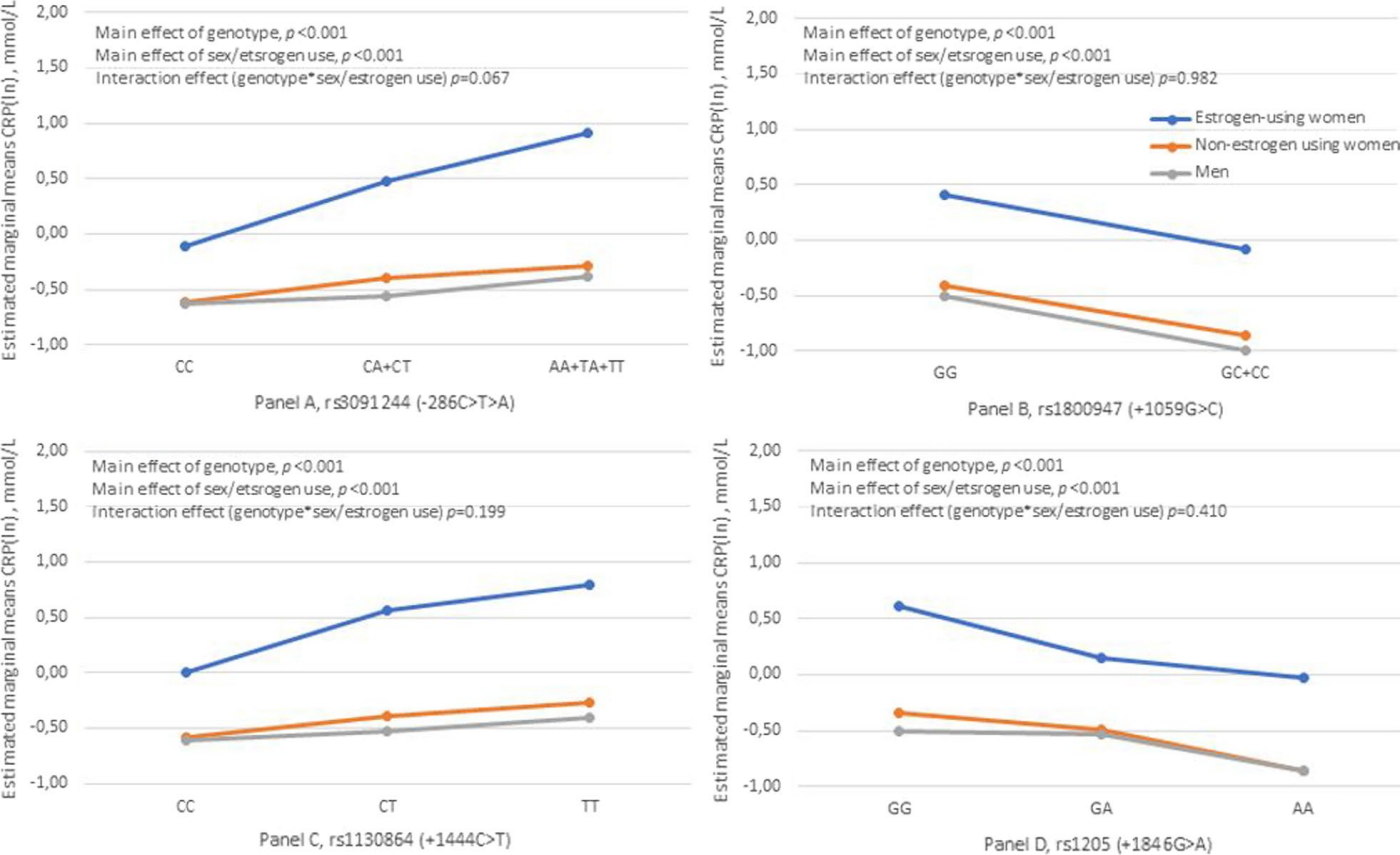
Estimated marginal means of C-reactive protein [CRP (ln)]. The *p*-values are based on 2-way ANOVA with genotype and sex/estrogen use as factors

CRP gene dosage for women (estrogen-users and non-estrogen users) and men for the four loci significantly associated with CRP (rs3091244, rs1800947, rs1130864, and rs1205), the CRP levels across the gene dosage separated by sex/estrogen use at three levels (i.e. women estrogen-users, women non-estrogen users, and men) are shown in Fig. 1. Main effects of genotype (all *p* < 0.001) and sex/estrogen use (all *p* < 0.001) were observed. No interaction effects were observed for genotype*sex/estrogen use (Fig. 1).

### Genotype associations to CRP after adjustment for risk factors

To assess the independent effect of each of the four genotypes on CRP(ln) in the total population, multiple regression analyses were performed with CRP (ln) as dependent variable and the common CVD risk factors BMI, LDL, triglycerides(ln), insulin(ln), systolic blood pressure, sex, estrogen use and genotype as covariates.

After adjusting for the risk factors in multivariate regression models (Table 3), all four SNPs remained significantly associated with the variation in CRP (all *p* < 0.001). The BMI showed the highest standardized beta coefficient to the variation in CRP followed by estrogen use and triglycerides. Sex contributed significantly to the variation in CRP only for rs1800947, where CRP was significantly lower in males than in females (*p* = 0.030). The explanatory power (R2 adj) and the magnitude of the beta coefficients were similar for all SNPs.

**Table 3:** Univariate and adjusted multiple linear regression models in 780 individuals of the LBA cohort

	rs3091244 (−286C>T>A)			rs1800947 (+1059G>C)			rs1130864 (+1444C>T)			rs1205 (+1846G>A)		
	Unstand b	Stand Beta	*p*	Unstand b	Stand Beta	*p*	Unstand b	Stand Beta	*p*	Unstand b	Stand Beta	*p*
Univariate models^1^												
Genotype	0.238	0.158	< 0.001	− 0.476	− 0.147	< 0.001	0.214	0.139	< 0.001	− 0.230	− 0.149	< 0.001
Multiple models^2^												
BMI	0.099	0.320	< 0.001	0.100	0.324	< 0.001	0.098	0.318	< 0.001	0.099	0.321	< 0.001
LDL	− 0.046	− 0.032	0.354	− 0.049	− 0.033	0.326	− 0.044	− 0.030	0.377	− 0.043	− 0.029	0.388
Triglycerides(ln)	0.478	0.187	< 0.001	0.469	0.183	< 0.001	0.482	0.188	< 0.001	0.483	0.189	< 0.001
Insulin(ln)	0.001	0.000	0.991	0.000	0.000	0.995	0.010	0.005	0.885	0.007	0.004	0.916
Systolic BP	− 0.003	− 0.032	0.443	− 0.001	− 0.013	0.760	− 0.003	− 0.032	0.437	− 0.003	− 0.033	0.425
Sex (F/M)	− 0.175	− 0.080	0.051	− 0.196	− 0.089	0.030	− 0.155	− 0.071	0.083	− 0.166	− 0.076	0.063
Estrogen use (No/yes)	0.713	0.263	< 0.001	0.718	0.265	< 0.001	0.718	0.265	< 0.001	0.718	0.265	< 0.001
Genotype	0.180	0.120	< 0.001	− 0.424	− 0.132	< 0.001	0.163	0.106	< 0.001	− 0.199	− 0.130	< 0.001

The C-reactive protein [CRP(ln)] was regressed on BMI, LDL, triglycerides(ln), insulin(ln), systolic blood pressure, sex, estrogen use and genotypes (rs3091244, rs1800947, rs1130864, rs1205). Abbreviations (unit) used: BMI = Body Mass Index (kg/m^2^); LDL = Low density lipoprotein (mmol/L); Tri = Triglycerides (mmol/L); Ins = Insulin (mU/L); BP = Blood pressure (mmHg); F/M = Female/Male; Unstand b = Unstandardized beta-coefficient; Stand Beta = Standardized beta-coefficient

^1^In the univariate models, CRP(ln) was regressed on the genotype, separately for each genotype

^2^In the multiple models, CRP(ln) was regressed on BMI, LDL, triglycerides(ln), insulin(ln), systolic blood pressure, sex, estrogen use and genotype, separately for each genotype. The adjusted R^2^ for the models were 0.250, 0.253, 0,247, and 0.253 for rs3091244, rs1800947, rs1130864, and rs1205, respectively

### Haplotype associations

Haplotype analysis revealed in 12 different haplotypes, whereof 5 were more frequent than 5% (Table 4). Haplotypes were presented in the order rs2794521, rs3091244, rs1800947, rs1130864, rs1205. The haplotype H1/ATGTG was the most frequent haplotype (31.3%), followed by H3/GCGCG (27.4%), H2/ACGCA (27.0%), H5/AAGCG (6.8%), H4/ACCCA (5.8%) and followed by additional haplotypes of less frequency. The haplotype analysis resulted in 6 different haplotype pairs (HP) with a frequency of more than 5%; HP2 (ATGTG/GCGCG) with 18.1%; HP1 (ACGCA/ATGTG) 16.0%; HP3 (ACGCA/GCGCG) 15.1%; HP4 (ATGTG/ATGTG) 10.6%; HP5 (ACGCA/ACGCA) 7.4% and HP10 (GCGCG/GCGCG) 6.9% (Table 4). In women, carriage of the haplotype H1/ATGTG was significantly associated to higher CRP levels (*p* < 0.001), while carriage of haplotype H2/ACGCA or H4/ACCCA were significantly associated to lower CRP levels (*p* = 0.047 and *p* < 0.001 respectively; Table 5). In men, carriage of the haplotype H4/ACCCA was significantly associated with lower CRP levels (*p* = 0.029; Table 5).

**Table 4:** Haplotype and haplotype pair frequencies in 780 individuals of the LBA cohort

Haplotype	Frequency, *n* (%)	Haplotype pair	Frequency, *n* (%)
H1: ATGTG	489 (31.3)	HP2: ATGTG/GCGCG	141 (18.1)
H3: GCGCG	428 (27.4)	HP1: ACGCA/ATGTG	125 (16.0)
H2: ACGCA	421 (27.0)	HP3: ACGCA/GCGCG	118 (15.1)
H5: AAGCG	106 (6.8)	HP4: ATGTG/ATGTG	83 (10.6)
H4: ACCCA	91 (5.8)	HP5: ACGCA/ACGCA	58 (7.4)
ACGCG	12 (0.8)	HP10: GCGCG/GCGCG	54 (6.9)
ACGTG	5 (0.3)	AAGCG/GCGCG	30 (3.8)
ATGCA	3 (0.2)	ACCCA/ATGTG	30 (3.8)
Other:	5 (0.3)	ACGCA/AAGCG	28 (3.6)
		ACGCA/ACCCA	24 (3.1)
		ATGTG/AAGCG	23 (2.9)
		ACCCA/GCGCG	23 (2.9)
		Other:	43 (5.5)

The order of the haplotypes is presented as rs2794521, rs3091244, rs1800947, rs1130864, rs1205. Haplotypes and haplotype pairs were named according to Eklund et al. [21] for haplotypes and haplotype pairs with a frequency > 5%

**Table 5:** Median CRP levels (Q1–Q3) in CRP haplotype carriers and non-carriers in the LBA cohort

Carriage of haplotype	Women total (*n* = 532)			Women estrogen users (*n* = 133)			Women non-estrogen users (*n* = 399)			Men total (*n* = 248)		
	CRP mg/L (*n*)		*p*	CRP mg/L (*n*)		*p*	CRP mg/L (*n*)		*p*	CRP mg/L (*n*)		*p*
	Median	Q1–Q3		Median	Q1–Q3		Median	Q1–Q3		Median	Q1–Q3	
H1												
ATGTG+	0.91 (272)	0.41–2.28	< 0.001	2.34 (77)	1.06–3.94	< 0.001	0.67 (195)	0.35–1.37	0.027	0.57 (134)	0.28–1.09	0.319
ATGTG−	0.59 (260)	0.29–1.19		0.87 (156)	0.53–2.24		0.52 (204)	0.26–0.99		0.46 (114)	0.22–1.26	
H3												
GCGCG+	0.66 (258)	0.32–1.56	0.340	1.17 (56)	0.60–2.91	0.105	0.55 (202)	0.29–1.14	0.733	0.54 (116)	0.24–1.09	0.640
GCGCG−	0.75 (274)	0.36–1.90		2.22 (77)	0.74–3.38		0.61 (197)	0.28–1.14		0.54 (132)	0.27–1.23	
H2												
ACGCA+	0.67 (251)	0.31–1.47	0.047	1.23 (57)	0.56–2.52	0.034	0.54 (194)	0.28–1.18	0.445	0.55 (112)	0.25–1.18	0.969
ACGCA−	0.74 (281)	0.40–1.93		1.87 (76)	0.81–4.01		0.59 (205)	0.30–1.11		0.54 (136)	0.24–1.15	
H5												
AAGCG+	0.84 (54)	0.46–2.20	0.137	3.04 (16)	1.01–5.06	0.027	0.60 (38)	0.38–1.20	0.697	0.57 (46)	0.25–1.18	0.690
AAGCG−	0.69 (478)	0.34–1.72		1.40 (117)	0.63–3.00		0.56 (361)	0.29–1.13		0.52 (202)	0.24–1.17	
H4												
ACCCA+	0.47 (69)	0.25–0.79	< 0.001	0.61 (13)	0.51–1.87	0.064	0.44 (56)	0.23–0.70	0.002	0.26 (20)	0.19–0.94	0.029
ACCCA−	0.75 (463)	0.37–1.94		1.77 (120)	0.76–3.24		0.61 (343)	0.31–1.28		0.57 (228)	0.26–1.24	

The order of the haplotypes is presented as rs2794521, rs3091244, rs1800947, rs1130864, rs1205. The *p*-values are based on Mann–Whitney U test. Haplotype carriers and haplotype non-carriers are marked as (+) or (−) respectively. Haplotypes H1–H5 were named according to Eklund et al. [21]

### Haplotype associations with CRP in estrogen and non-estrogen users

**Fig. 2:**
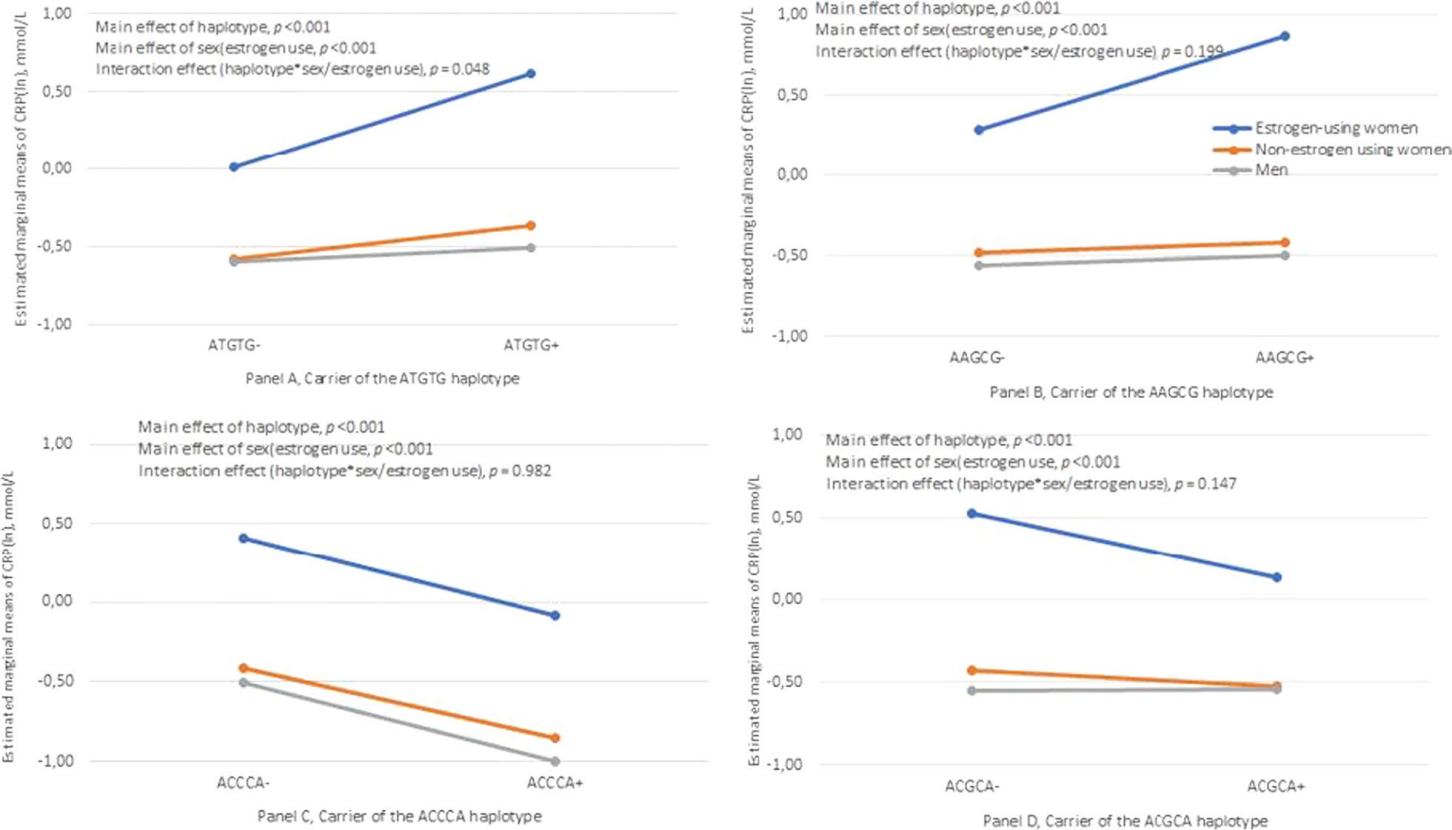
Estimated marginal means of C-reactive protein [CRP (ln)]. The *p*-values are based on 2-way ANOVA with haplotype and sex/estrogen use as factors

After stratification of women into estrogen users and non-estrogen users, carriage of the H1/ATGTG haplotype was significantly associated with higher CRP levels, and there was a more prominent difference between carriers compared to non-carriers in the estrogen user group (Median EU non-carriers vs. carriers: 0.87–2.34 mg/L, *p* < 0.001; Median NEU non-carriers vs. carriers: 0.52–0.67 mg/L, *p* = 0.027; Table 5). Analyzed by 2-way ANOVA, an interaction effect between H1/ATGTG haplotype and sex/estrogen use was observed (*p* = 0.048; Fig. 2A). Carriers of haplotype H5/AAGCG also had significantly higher CRP levels compared to non-carriers in female estrogen users (Median EU non-carriers vs. carriers: 1.40–3.04 mg/L, *p* = 0.027; Table 5), which could not be observed in non-estrogen users.

The trend of H4/ACCCA haplotype carriage and association with lower CRP levels was similar even after stratification of women into estrogen users and non-estrogen users, although statistically significant only in the non-estrogen user group (Median NEU carriers vs. non-carriers: 0.44–0.61 mg/L, *p* = 0.002; Table 5). Significantly lower CRP level was found in female carriers of the H2/ACGCA haplotype in the estrogen user group (Median carriers vs. non-carriers: 1.23–1.87 mg/L, *p* = 0.034; Table 5) but not in the non-estrogen user group. No interaction effects between H5/AAGCG, H4/ACCCA or H2/ACGCA haplotype and sex/estrogen use analyzed by 2-way ANOVA could be observed (Fig. 2B–D).

### Associations between CRP, genotype or haplotype and Intima-Media Thickness (cIMT)

Only minor variations in cIMT (min–max in total population: 0.33–0.82 mm) was observed. There was a significant correlation between cIMT and CRP(ln) in female estrogen users (*r* = 0.194, *p* = 0.026) but no significant correlation in female non-estrogen users or men. Significant associations were observed between cIMT levels and rs1800947 genotype in women total group and in non-estrogen users. In addition, association between cIMT and genotype rs1130864 in female estrogen users were found (Additional file 1: Table S1).

As for genotype, only minor variations in cIMT between genotypes of the different haplotypes was observed. Interestingly, in estrogen users, carriage of the haplotype H1/ATGTG or H2/ACGCA were significantly associated with cIMT, which was in line with elevated and reduced CRP levels respectively, in these groups (H1/ATGTG Median carriers vs non-carriers: 0.49–0.47 mm; *p* = 0.002; H2/ACGCA Median carriers vs non-carriers: 0.47–0.49; *p* = 0.028; Additional file 1: Table S2; Table 5). However, although we found a significant association in the total women group and in the non-estrogen user group between H4/ACCCA and cIMT (Total women Median carriers vs non-carriers: 0.51–0.49; *p* = 0.002; NEU Median carriers vs non-carriers: 0.51–0.49; *p* = 0.010; Additional file 1: Table S2), the trend was opposite compared to CRP levels (Additional file 1: Table S2; Table 5), why it is difficult to draw any clear conclusions on the role of these genotypes or haplotypes and its effects on preclinical signs of atherosclerosis. No associations between carriage of the additional haplotypes H3/GCGCG or H5/AAGCG and cIMT in women, regardless of estrogen use or not, were observed. No association between cIMT and genotype or haplotype in men were found.

## Discussion

Elevated level of CRP has been suggested as a strong predictor of cardiovascular events and genetic variations in SNPs in the CRP gene has in different studies been associated with higher or lower CRP levels [18, 20, 34–39]. One of the aims of the present study was to elucidate the relationship between CRP genotype and signs of early atherosclerosis in the Swedish Lifestyle, Biomarkers and Atherosclerosis (LBA) cohort, which consists of young, healthy non-smoking individuals. Elevated cIMT is an indicator of hypertrophy in the intima and media layers of the vessel wall and cIMT is generally considered to be a sensitive and reproducible measurement for the quantification of early atherosclerosis [40].

In the present study, we observed that the haplotypes H1/ATGTG and H2/ACGCA were significantly associated with cIMT, which was interestingly also associated to higher and lower CRP levels respectively, in female estrogen users. In addition, the correlation between CRP and cIMT levels were interestingly only found significant in female estrogen users. However, the significant differences observed in cIMT levels between carriers and non-carriers were very small and not clinically relevant, why this data should therefore be interpreted cautiously. Yet, this gives an implication for future longitudinal and larger follow-up studies on the role of estrogen use and atherosclerosis, since small subgroups is a limitation of the present study. However, in two Finnish cross-sectional studies with healthy individuals performed on the same SNPs as in the present study, no association between cIMT and genetic variations in the CRP locus were found [21, 22]. However, they found that the genotypes rs3091244, rs1130864 and rs1205 were significantly associated with arterial stiffness measures [21] and the SNP rs1130864 was significantly associated with stiffness variables [22] in male individuals, but not in females. While cIMT is a measure of structural changes of the vessel wall, the stiffness variables are functional indicators of the elasticity of large arteries, which make the study results precarious to compare as they address different properties of the artery. The clinical relevance of indices is difficult to evaluate, since an index is a combination of all included variables, whereas cIMT is an early noticeable manifestation, even if very thin.

Another aim of the present study was to evaluate the association between genotype and haplotype with CRP levels in the LBA cohort. We found that the SNPs rs3091244, rs1800947, rs1130864 and rs1205 polymorphisms in the *CRP* locus were significantly associated with higher or lower CRP levels in women. In men, we found a significant association between CRP levels and the SNP rs1800947 only. Several genetic association studies have been performed on the association between CRP levels and genotype, on different cohorts and with different outcomes [18, 20–22, 36–39]. As in the present study, the studies made by Eklund et al. [21] and Kettunen et al. [22] focused on healthy individuals as well as the same SNPs. However, the results from the three studies differ. Eklund et al. [21] observed a significant association with CRP levels for all of the five SNPs investigated in the present study in female individuals and to four of the five SNPs in men, only the rs2794521 polymorphism did not reach statistical significance [21]. Kettunen et al. [22] observed a significant association only with the rs1800947 polymorphism in women and a significant association in men for the SNPs rs2794521 and rs1800947 only.

We also found significant associations between the haplotypes H1/ATGTG, H2/ACGCA and H4/ACCCA and CRP levels in women and only significant associations between CRP levels and the H4/ACCCA haplotype in men. Carriage of the haplotype H1/ATGTG was significantly associated with higher CRP levels in women but not in men, which is in line with Eklund et al. [21], although they also found a significant association in men. Kettunen et al. [22] found a non-significant but borderline trend of association to elevated CRP levels for carriers of the H1/ATGTG haplotype in women, but no association in men. On the other hand, carriers of the H4/ACCCA haplotype was significantly associated with lower CRP levels in both women and men in the present study. Kettunen et al. [22] and Eklund et al. [21] found a significant association between H4/ACCCA genotype and CRP levels in women and men respectively.

The specific reason to the differences between the present study and the studies by Eklund et al. and Kettunen et al. [21, 22] is very difficult to elucidate and beyond the focus of the present investigation and remains elusive. There are however relevant demographic and environmental differences between the present cohort and the cohorts used by Eklund et al. [21] and Kettunen et al. [22] that may have resulted in different outcome of the three studies. Although genetic drift has been proposed in Sweden, Swedish individuals have been suggested to be more genetically similar to Germans and British individuals compared to Finns [41, 42], allele frequencies in the total population presented by Eklund et al. [21] and Kettunen et al. [22] were relatively similar as in the present study. In addition, age is a significant difference between the three cohorts, that may have caused the observed differences. The Swedish LBA cohort contains healthy individuals from young adults 18–25.9 years, while the two Finnish studies used healthy individuals (≤ 18 years) or older (> 30 years, mean age 58.2 years) population groups [21, 22]. The participants in the present study were only slightly older than the individuals in the study by Eklund et al. [21], and interestingly, the comparisons between CRP and genotype for women in the present study were slightly more like the results made by Eklund et al. [21] compared to Kettunen et al. [22]. The latter study was performed on an older cohort, probably more affected by different age-related exposures, such as smoking. Smoking has been pointed out as an important factor that correlates significantly in men with CRP levels [25]. The LBA cohort consisted of non-smoking individuals, while the two Finnish cohorts contained smokers [21, 22]. Other age-related exposures, such as estrogen exposure may also explain the differences between the present study and the study made by Eklund et al. and Kettunen et al. [21, 22]. Several of the female participants in the study by Kettunen et al. [22] were most likely menopausal. Although CRP levels are in general considered to be higher in female individuals compared to male individuals (for example Pai et al. [43]), Kettunen et al. [22] did not find any difference in CRP levels between men and women, which supports this hypothesis. In the LBA cohort, some of the women were utilizing estrogen containing contraceptives and due to the fact that estrogen is associated with higher CRP levels [24, 25], we hypothesized that it might be of importance to stratify the effect of genotype on CRP levels upon estrogen use. In the present study, female estrogen users had significantly higher median CRP levels compared to female non-estrogen users, which may suggest that it is of importance to control for estrogen levels in similar studies. Although female individuals in the present study were stratified for estrogen use, the genotype effect sustained throughout the four significant genotypes, with slight differences. The rare alleles of the SNPs rs3091244 and rs1205 were associated with higher and lower CRP levels respectively, in both estrogen user and non-estrogen user groups. However, the association between CRP and female estrogen users for the SNP rs1800947 and female non-estrogen users for the SNP rs1130864 were of borderline but not significant after stratification dependent on estrogen use, which may be dependent on few individuals in the different subgroups, which is a clear limitation of the present study. Another limitation is that after stratification into genotype groups, the genotype group sizes vary and the power to detect significant mean differences varies accordingly. Effect sizes on genotype level may become underpowered due to small numbers in the rare allele groups. Anyway, due to the fact that estrogen is associated with higher CRP levels, our data indicate that it may be of importance to stratify fertile female individuals upon estrogen use in future similar studies.

## Conclusions

Collectively, we have shown strong associations between CRP levels and the SNPs in the *CRP* gene rs3091244, rs1800947, rs1130864, and rs1205 in women, but only significant association with the SNP rs1800947 in men. Female carriers of the H1/ATGTG haplotype had significantly higher CRP levels than non-carriers, and female estrogen users had the highest CRP levels, which was also associated with a small but significantly higher cIMT level, although probably clinically irrelevant. Carriage of H1/ATGTG haplotype could therefore be a risk factor for elevated CRP levels and possibly also a small increment of cIMT levels in female estrogen users. However, future and larger studies are warranted in order to elucidate the role of CRP levels in relation to estrogen, genotype, haplotype and early signs of atherosclerosis to see if these findings are generalizable.

## Supplementary Information

**Additional file 1.** Median cIMT levels (Q1-Q3) in the LBA cohort in relation to SNPs (Table S1) and haplotypes (Table S2) in the* CRP* gene.

## Data Availability

Data sets generated and analysed in the current study are not public available due to research subject confidentiality but are aware in a de-identified form from the corresponding author and PI upon reasonable request.

## Abbreviations

CRP: C-reactive protein
cIMT: Carotid intima media thickness
PCR: Polymerase chain reaction
hs: High-sensitive
CVD: Cardiovascular disease
VCAM: Vascular cell adhesion protein
ICAM: Intracellular adhesion molecule
IL: Interleukin
MAC: Membrane attack complex
CAC: Carotid artery compliance
CAE: Carotid artery elasticity
SNP: Single nucleotid polymorphism
LBA study: Lifestyle, Biomarkers and Atherosclerosis study
EU: Estrogen users
NEU: Non-estrogen users
DNA: Deoxyribonucleic acid
BMI: Body mass index
LDL: Low-density lipoprotein
HDL: High-density lipoprotein
