# Supplementary material for: CRP levels are significantly associated with CRP genotype and estrogen use in The Lifestyle, Biomarker and Atherosclerosis (LBA) study
Source: BMC Cardiovasc Disord. 2022 Apr 15;22:170. doi: 10.1186/s12872-022-02610-z (PMC9013148; doi:10.1186/s12872-022-02610-z)
Supplement: Supplementary file 1 — Additional file 1. Median cIMT levels (Q1-Q3) in the LBA cohort in relation to SNPs (Table S1) and haplotypes (Table S2) in the CRP gene. [file 12872_2022_2610_MOESM1_ESM.docx]

**Table S1. Median cIMT levels (Q1-Q3) in the LBA cohort in relation to *CRP* gene SNPs.**

|  | **Women  Total (*n*= 524)** | | | **Women**  **Estrogen users (*n*= 132)** | | | **Women Non-estrogen users (*n*=392)** | | | **Men  Total (*n*= 246)** | |  |
| --- | --- | --- | --- | --- | --- | --- | --- | --- | --- | --- | --- | --- |
|  | **cIMT mm (n)** |  | ***p*** | **cIMT mm (n)** |  | ***p*** | **cIMT mm (n)** |  | ***p*** | **cIMT mm (n)** |  | ***p*** |
|  | **Median** | **Q1-Q3** |  | **Median** | **Q1-Q3** |  | **Median** | **Q1-Q3** |  | **Median** | **Q1-Q3** |  |
| **rs2794521** (-717A>G) |  |  |  |  |  |  |  |  |  |  |  |  |
| AA | 0.50 (268) | 0.46-0.53 | 0.148 | 0.48 (76) | 0.46-0.53 | 0.610 | 0.50 (192) | 0.47-0.53 | 0.195 | 0.50 (131) | 0.47-0.54 | 0.451 |
| AG | 0.49 (214) | 0.45-0.52 |  | 0.48 (44) | 0.45-0.51 |  | 0.49 (170) | 0.45-0.53 |  | 0.50 (102) | 0.46-0.53 |  |
| GG | 0.49 (42) | 0.47-0.55 |  | 0.49 (12) | 0.46-0.51 |  | 0.49 (30) | 0.47-0.55 |  | 0.50 (13) | 0.47-0.54 |  |
|  |  |  |  |  |  |  |  |  |  |  |  |  |
| **rs3091244** (-286C>T>A) |  |  |  |  |  |  |  |  |  |  |  |  |
| CC | 0.49 (206) | 0.46-0.53 | 0.229 | 0.48 (45) | 0.46-0.50 | 0.313 | 0.49 (161) | 0.46-0.54 | 0.491 | 0.49 (80) | 0.46-0.53 | 0.268 |
| CA+CT | 0.49 (251) | 0.45-0.53 |  | 0.49 (63) | 0.45-0.52 |  | 0.49 (188) | 0.46-0.53 |  | 0.50 (120) | 0.47-0.54 |  |
| AA+TA+TT | 0.51 (67) | 0.47-0.54 |  | 0.50 (24) | 0.46-0.56 |  | 0.51 (43) | 0.48-0.53 |  | 0.50 (46) | 0.47-0.55 |  |
|  |  |  |  |  |  |  |  |  |  |  |  |  |
| **rs1800947** (+1059G>C) |  |  |  |  |  |  |  |  |  |  |  |  |
| GG | 0.49 (456) | 0.46-0.53 | 0.003 | 0.48 (119) | 0.45-0.52 | 0.176 | 0.49 (337) | 0.45-0.53 | 0.010 | 0.50 (226) | 0.47-0.54 | 0.401 |
| GC+CC | 0.51 (68) | 0.47-0.56 |  | 0.49 (13) | 0.48-0.55 |  | 0.51 (55) | 0.47-0.56 |  | 0.51 (20) | 0.47-0.55 |  |
|  |  |  |  |  |  |  |  |  |  |  |  |  |
| **rs1130864** (+1444C>T) |  |  |  |  |  |  |  |  |  |  |  |  |
| CC | 0.49 (250) | 0.45-0.53 | 0.187 | 0.47 (56) | 0.43-0.50 | 0.007 | 0.49 (194) | 0.45-0.54 | 0.504 | 0.49 (111) | 0.46-0.53 | 0.182 |
| CT | 0.49 (220) | 0.46-0.53 |  | 0.50 (59) | 0.47-0.53 |  | 0.49 (161) | 0.46-0.53 |  | 0.50 (106) | 0.47-0.53 |  |
| TT | 0.50 (54) | 0.47-0.53 |  | 0.48 (17) | 0.46-0.56 |  | 0.51 (37) | 0.48-0.53 |  | 0.51 (29) | 0.48-0.57 |  |
|  |  |  |  |  |  |  |  |  |  |  |  |  |
| **rs1205** (+1846G>A) |  |  |  |  |  |  |  |  |  |  |  |  |
| GG | 0.49 (228) | 0.46-0.53 | 0.948 | 0.49 (67) | 0.46-0.53 | 0.292 | 0.49 (161) | 0.46-0.53 | 0.834 | 0.50 (119) | 0.47-0.55 | 0.550 |
| GA | 0.49 (234) | 0.46-0.53 |  | 0.49 (48) | 0.45-0.51 |  | 0.50 (186) | 0.46-0.53 |  | 0.49 (106) | 0.47.0.52 |  |
| AA | 0.49 (62) | 0.44-0.53 |  | 0.47 (17) | 0.44-0.53 |  | 0.50 (45) | 0.44-0.54 |  | 0.51 (21) | 0.44-0.53 |  |

The *p*-values are based on Kruskal Wallis test (if three or more groups) or Mann-Whitney U test (if two groups).

**Table S2. Median cIMT levels (Q1-Q3) in CRP haplotype carriers and non-carriers in the LBA cohort.**

|  | **Women**  **Total (*n*= 532)** | | | **Women**  **Estrogen users (*n*= 133)** | | | **Women**  **Non-estrogen users (*n*= 399)** | | | **Men**  **Total (*n*= 248)** | | |
| --- | --- | --- | --- | --- | --- | --- | --- | --- | --- | --- | --- | --- |
| **Carriage of haplotype** | **cIMT mm (n)** |  | ***p*** | **cIMT mm (n)** |  | ***p*** | **cIMT mm (n)** |  | ***p*** | **cIMT mm (n)** |  | ***p*** |
|  | **Median** | **Q1-Q3** |  | **Median** | **Q1-Q3** |  | **Median** | **Q1-Q3** |  | **Median** | **Q1-Q3** |  |
| H1 |  | |  |  | |  |  | |  |  | |  |
| ATGTG+ | 0.49 (270) | 0.46-0.53 | 0.113 | 0.49 (76) | 0.47-0.54 | 0.002 | 0.49 (194) | 0.46-0.53 | 0.877 | 0.50 (133) | 0.47-0.54 | 0.327 |
| ATGTG- | 0.49 (254) | 0.45-0.53 |  | 0.47 (56) | 0.43-0.50 |  | 0.49 (198) | 0.45-0.54 |  | 0.49 (113) | 0.46-0.53 |  |
|  |  | |  |  | |  |  | |  |  | |  |
| H3 |  | |  |  | |  |  | |  |  | |  |
| GCGCG+ | 0.49 (256) | 0.46-0.52 | 0.151 | 0.48 (56) | 0.46-0.51 | 0.560 | 0.49 (200) | 0.46-0.53 | 0.172 | 0.50 (115) | 0.46-0.54 | 0.332 |
| GCGCG- | 0.50 (268) | 0.46-0.53 |  | 0.48 (76) | 0.46-0.53 |  | 0.50 (192) | 0.47-0.53 |  | 0.50 (131) | 0.47-0.54 |  |
|  |  | |  |  | |  |  | |  |  | |  |
| H2 |  | |  |  | |  |  | |  |  | |  |
| ACGCA+ | 0.49 (245) | 0.45-0.53 | 0.113 | 0.47 (56) | 0.44-0.51 | 0.028 | 0.49 (189) | 0.45-0.53 | 0.500 | 0.49 (111) | 0.46-0.52 | 0.103 |
| ACGCA- | 0.49 (279) | 0.46-0.53 |  | 0.49 (76) | 0.46-0.53 |  | 0.49 (203) | 0.46-0.53 |  | 0.50 (135) | 0.47-0.55 |  |
|  |  | |  |  | |  |  | |  |  | |  |
| H5 |  | |  |  | |  |  | |  |  | |  |
| AAGCG+ | 0.50 (53) | 0.43-0.52 | 0.408 | 0.47 (16) | 0.42.0.52 | 0.164 | 0.50 (37) | 0.44-0.53 | 0.923 | 0.40 (45) | 0.46-0.55 | 0.977 |
| AAGCG- | 0.49 (471) | 0.46-0.53 |  | 0.48 (116) | 0.46-0.52 |  | 0.49 (355) | 0.46-0.53 |  | 0.50 (201) | 0.47-0.54 |  |
|  |  | |  |  | |  |  | |  |  | |  |
| H4 |  | |  |  | |  |  | |  |  | |  |
| ACCCA+ | 0.51 (68) | 0.47-0.56 | 0.002 | 0.49 (13) | 0.48-0.55 | 0.176 | 0.51 (55) | 0.47-0.56 | 0.010 | 0.51 (20) | 0.47-0.55 | 0.401 |
| ACCCA- | 0.49 (456) | 0.46-0.53 |  | 0.48 (119) | 0.45-0.52 |  | 0.49 (337) | 0.45-0.53 |  | 0.50 (226) | 0.47-0.54 |  |
|  |  | |  |  | |  |  | |  |  | |  |
|  |  | |  |  | |  |  | |  |  | |  |

The order of the haplotypes is presented as rs2794521, rs3091244, rs1800947, rs1130864, rs1205. The *p*-values are based on Mann-Whitney U test. Haplotype carriers and haplotype non-carriers are marked as (+) or (-) respectively. Haplotypes H1-H5 were named according to Eklund et al [21].
